# The Patient Voice Is In: Building Systems of Diagnostic Care That Listen and Learn; A Response to Recent Commentaries

**DOI:** 10.34172/ijhpm.10223

**Published:** 2026-08-25

**Authors:** Kathryn M. McDonald, Kelly T. Gleason, Anushka Jajodia, Helen Haskell, Vadim Dukhanin

**Affiliations:** ^1^School of Nursing, Johns Hopkins University, Baltimore, MD, USA.; ^2^Division of General Internal Medicine, Department of Medicine, Johns Hopkins University, Baltimore, MD, USA.; ^3^Mothers Against Medical Error, Columbia, SC, USA.; ^4^Department of Health Policy and Management, Bloomberg School of Public Health, Johns Hopkins University, Baltimore, MD, USA.

 The thoughtful commentaries by Schmidt and Riposo, Cosby, Chen et al, and Ranji and Rosner on “Achieving Diagnostic Excellence: Roadmaps to Develop and Use Patient-Reported Measures With an Equity Lens” (Roadmaps article) contribute important perspectives on incorporating patient voice in improving diagnosis for all.^1^ Three converging developments mark apivotal moment for operationalizing patient-reported diagnostic excellence: maturation of artificial intelligence (AI) technologies that enhance patient reporting, establishment of coordinating infrastructure, and proliferation of real-world implementation examples across diverse clinical contexts and international settings. Here, we first situate our Roadmaps article within a three-paper series and then address the commentators’ perspectives through three interconnected themes that together demonstrate how patient-reported diagnostic excellence is transitioning from concept to practice.

## Situating the Roadmaps Article Within a Three-Paper Series

 Our work developing roadmaps represents the third component of a logical sequence addressing complementary questions about patient-reported diagnostic excellence. The first paper in this series comprised a scoping review that identified diagnostically relevant domains for capturing patient-reported outcomes and experiences, establishing WHAT should be measured.^2^ The second paper presented the Framework for Patient-reported Measurement Opportunities of Diagnostic Excellence, which organized patient diagnostic journeys into a taxonomy based on timing, setting, and diagnostic care utilization and specified WHEN, WHERE, and FROM WHOM these domains can be assessed.^3^ The FROM WHOM envisioned that measurement opportunities encompass voices from all, including those whose diagnostic journeys are typically invisible to health systems.

 The Roadmaps article then addressed HOW to develop and implement patient-reported measures (PRMs) strategically by presenting seven exemplar goals that PRMs can achieve for diagnostic excellence. For each goal, we provided measure concepts and use cases. We presented visual roadmaps guiding diverse stakeholders through PRM stages from concept to goal, indicating synergies to leverage, challenges to overcome, and projected timeframes. This research led to a VISION of patient-reported diagnostic excellence that is co-driven by patients, optimizes experience and outcomes equitably across settings, and expands diagnosis beyond disease codes and labels to its value for holistic and informed living for the patient.

 Collectively, the three-paper series amplified the patient voice: WHAT diagnostically relevant domains patients might report, FROM WHOM encompassing populations experiencing diagnostic journeys to ensure WHEN and WHERE decisions capture voices from all patients, and HOW sets up measurement systems strategically toward the VISION.

## AI-Enhanced Patient Reporting: Capabilities and Converging Trends

 Ranji and Rosner astutely identify AI integration as central to accelerating our roadmaps and introduce the concept of “diagnostic wayfinding,” which leverages electronic health record data and AI to characterize diagnostic journeys.^4^ Since our Expert Convening in June 2021, several AI-enabled capabilities have emerged as significant drivers in advancing patient reporting of diagnostic excellence across the four PRM stages in our roadmaps (Table).

**Table:** AI-Enhanced Capabilities Across Roadmap Stages of Patient-Reported Measurement for Diagnostic Excellence

**Development and Endorsement**^a^** Stages**	**Implementation and Scaling Stage**		**Acting Upon the Measure Stage**
*AI-enhanced measure construction and validation:* Narrative analysis Granular domain discovery Question and item generation Synthetic-cohort pre-validation Multilingual and cultural item adaptation	*AI-enhanced administration and collection:* Adaptive voice recognition Interactive voice response systems Multimodal chatbots Dynamic questionnaire personalization	*AI-enhanced analysis and use in workflow:* Unstructured-to-structured data mapping Diagnostic wayfinding Audience-tailored score interpretation Narrative-centric visualization	*AI-enhanced real-time response facilitation:* Just-in-time tailored education Real-time safety alerts and triage Patient-clinician communication support Disengagement detection and outreach responses

Abbreviation: AI, artificial intelligence. ^a^AI technologies support technical validation and evidence generation for measure endorsement but do not address the equally critical cultural, social, and organizational dimensions. Stakeholder acceptance, cultural change, and organizational readiness require coordinated human-centered processes rather than AI-enabled technologies (See Cosby commentary).

 These AI-enhanced capabilities are well-positioned to enable several converging trends that fundamentally reshape PRM benefits beyond technical acceleration:

- First, PRMs collected for organizational quality improvement could also trigger safety alerts through instantaneous AI-enabled collection and analysis, creating an imperative rather than merely a possibility for real-time response for individual patient care and protection, while still simultaneously supporting systematic learning cycles. Similarly, PRMs collected at population levels for diagnostic continuity tracking or research purposes could also be analyzed and used more rapidly for individual interventions.
- Second, and most critically, patient engagement and patient reporting are merging into mutually reinforcing processes. Real-time AI capabilities create patient expectations for immediate action from those collecting PRMs. Patients increasingly anticipate that reporting their experience or outcomes will trigger timely responses, fundamentally shifting implicit agreements between those providing their data (patients) and those in a position to act upon the data (organizations collecting PRMs). Given that diagnosis occurs over time, the ability to move from delayed aggregation and retrospective analysis of PRMs to dynamic interaction and real-time intervention has significant value in building better systems of diagnosis that rely on patient reporting.
- Third, the traditional boundary between structured PRMs and unstructured patient narratives is dissolving. AI technologies can now map patient voices captured in narratives onto PRM domains, reinforcing our use of “PRMs” for diagnostic excellence as an inclusive term encompassing outcome measures, experience measures, and patient stories.

However, as AI capabilities enhance the flow and analysis of PRMs, the critical bottleneck will shift to routing insights for action. With finite capacity for diagnostic tests, appointments, and care adjustments, sophisticated triage systems must determine which patient reports require immediate response versus aggregation for quality improvement.

## From Theory to Practice: Real-World Implementation Examples Around the Globe

 Chen and colleagues’ application of our roadmaps to prenatal screening and testing (PreST) exemplifies the concrete application that Schmidt and Riposo sought, showing how condition-specific context adaptation can maintain core principles and illustrate the road-mapping process.^5,6^ Chen and colleagues developed a 12-year roadmap specific to PreST that incorporates both the goal for evaluating routine screenings and the goal for supporting patient storytelling, demonstrating how multiple goals can be pursued synergistically.

 Schmidt and Riposo expressed concern about long timeframes for PRM-enabled diagnostic excellence potentially dissuading organizations from engaging in measurement and requested prioritization guidance for which measure concepts to develop first.^6^ Current fieldwork for measure development and use is underway where diagnostic safety concerns intersect with measurement feasibility:

- Patient-Report to IMprove Diagnostic Excellence in Emergency Department settings (PRIME-ED) illustrates accelerated PRM development from conception through multi-site implementation and learnings across four US health systems, and additional adaptation of this measure approach at Karolinska Institute in Sweden.^7,8^
- The Patient-Reported Experience and Outcomes of Safety in Primary Care (PREOS-PC) demonstrates progression in measurement, having evolved into a compact version with validated multilingual cross-country adaptations of this instrument set.^9^
- The Designing Discharge after Emergency Care (D-DEC) project adapted the Patient-Reported Outcome Measure for Emergency Department care (PROM-ED), a research and quality improvement questionnaire, to deliver targeted support at critical discharge transition points in Canada.^10^

Additional measures are emerging at the measurement development stage:

- Patient-Reported Outcome Measures of Timely Experience of Diagnosis for Cancer (PROMOTE-Dx), assessing both pre-health system and health system factors, completed field testing and is ready for endorsement.^11^
- The Patient-Reported Experience Measure for Prenatal Screening (PREM-PS), advancing within Chen and colleagues’ PreST roadmap, achieved validation across Canadian French and English populations.^5,12^
- The Active-Empathic Listening Scale (AELS) achieved validation for measuring clinician listening behaviors.^13^

Pre-validation efforts for nationwide surveys are underway in both Japan and the United States, with the latter demonstrating feasibility of eliciting diagnostic problem and mistake experiences across diverse sociodemographic populations.^14^ A parallel development strand embeds structured narrative elicitation directly into clinical workflows, with OurDX, a pre-visit online engagement tool, capturing diagnostic safety opportunities across multiple settings and Safer Dx Patient Instrument for post-visit note review tested in an adult primary care cohort.^15,16^

## Infrastructure and Coordination: Building Systematic Capacity

 Cosby’s commentary emphasizes coordinating infrastructure and systematic approaches as essential to moving from isolated projects to sustained progress.^17^ Her call for a coordinating center to convene experts, debate methods and priorities, analyze and summarize lessons, disseminate best practices and tools, and coordinate global efforts resonates deeply with our vision and research. We can now report progress on this front:

- The Center for Patient-Reported Measures of Diagnostic Excellence has been established to serve as a nexus for researchers, patients, practitioners, and the public in shared concerns about diagnostic safety, quality, accountability, and equity. The center advances PRMs from development to use in the shared quest for diagnostic excellence galvanized by measures that matter.
- The University of California, San Francisco Coordinating Center for Diagnostic Excellence offers complementary infrastructure for coordination, serving as a national coordinating entity and operating through three programs: a Learning Hub, an Engagement Hub, and an Action Incubator.
- The US National Quality Forum has designated diagnostic excellence as a measurement priority, highlighting the need for PRMs that improve diagnosis for every patient.^18^ The report emphasizes actionable measures incorporating the patient voice to drive diagnostic quality improvement and accountability for all people.

## The Patient Voice Is In

 In 2013, McDonald et al wrote “The Patient Is In” about patient involvement strategies for diagnostic error mitigation.^19^ More than a decade later, we can say the patient *voice* is in. We can hear that voice because we have established WHAT to measure, WHEN and WHERE to assess it, FROM WHOM and HOW, with measures advancing a VISION. The patient voice must remain core to shaping and validating all measurement tools and their uses to advance diagnostic excellence. The voice is on the cusp of being amplified by AI technologies that enable real-time collection, analysis, interpretation, response, and continuous learning about how best to build systems of diagnostic care with patients and their care partners (families and friends). These same technologies can dissolve traditional boundaries between aggregate retrospective analysis and individual-response routing, between structured surveys and narratives. Yet they simultaneously expose bottlenecks – in cultural and organizational readiness and in finite operational capacity – and set new expectations for patient engagement and real-time action. With infrastructure and active collaborations gaining ground for systematic coordination and designation of PRMs for diagnostic excellence as a measurement priority, the momentum will keep coming from implementation teams across the globe deploying measures in routine care settings, advancing along roadmaps enriched by these four commentaries.

## Acknowledgements

 We gratefully acknowledge patients, families and their advocates who voice concerns about patient safety, quality and equity as they seek improvements in health systems for themselves and others.

## Disclosure of artificial intelligence (AI) use

 AI tools (Claude and ChatGPT) were used for editing language and improving grammar in all sections.

## Ethical issues

 Not applicable.

## Conflicts of interest

 Authors declare that they have no conflicts of interest.
